# A new species of *Notomastus* (Annelida, Capitellidae) from southern China, with remarks on its morphology and distribution

**DOI:** 10.3897/zookeys.946.50662

**Published:** 2020-07-06

**Authors:** Jun-Hui Lin, María E. García-Garza, Ming-Xin Lyu, Jian-Jun Wang

**Affiliations:** 1 Third Institute of Oceanography, Ministry of Natural Resources, 178 Daxue Road, Xiamen 361005, China Third Institute of Oceanography Xiamen China; 2 Universidad Autónoma de Nuevo León, Facultad de Ciencias Biológicas, Laboratorio de Biosistemática, Apartado Postal 5 “F”, San Nicolás de los Garza, Nuevo León, México Universidad Autónoma de Nuevo León San Nicolas de los Garza Mexico; 3 State Key Laboratory of Marine Environmental Science, College of Ocean and Earth Sciences, Xiamen University, South Xiang’an Road, Xiamen 361102, China Xiamen University Xiamen China

**Keywords:** coastal waters, Polychaeta, sequences analysis, southern China, systematics

## Abstract

The genus *Notomastus* is frequently encountered in Chinese waters. However, its species richness is poorly understood. In this study, a *Notomastus* species obtained from Xiamen Bay, southern China, was described and illustrated as a new species (*N. sunae***sp. nov.**), based on morphological and molecular analyses. The new species is characterized by having uniramous chaetiger 1, the presence of palpode and eyespots on prostomium, chaetiger 11 with notopodial capillaries and neuropodial hooded hooks, and notopodial lobes with simple epithelial extensions on far posterior abdomen. With additional specimens collected from several localities along the southern coasts of China, the morphology and geographical distribution of the new species are discussed. A key is also provided for *Notomastus* species with neuropodial hooks in thoracic chaetiger 11.

## Introduction

Polychaetes of the family Capitellidae, which is among the most common families in marine surveys, are distributed at depths from the intertidal to abyssal zones (Hernández-Alcántara and Solís-Weiss 1998; Blake 2000). Of the 43 known genera, *Notomastus* is the most species-rich genus in the family Capitellidae and includes 43 described species worldwide (García-Garza et al. 2019). *Notomastus* was initially erected by Sars (1851) for the type species *N. latericeus* from Norway, a capitellid bearing an achaetous peristomium, 11 thoracic chaetigers with only capillaries, and an abdomen having only chaetigers with hooded hooks. However, newly described species have added morphological variability to the genus and the generic diagnosis became increasingly obscure. For instance, Hartman (1960) described a *Notomastus* species with neuropodial hooks in last thoracic chaetiger, which is not found in most members of the genus. To clarify the taxonomic boundary of the genus, an emended generic diagnosis was proposed over time by several authors. Ewing (1982) and later Blake (2000) proposed a broader definition to include species from morphologically similar genera, such as *Paraleiocapitella*, *Dodecaseta*, and *Rashgua*. However, Green (2002) preferred to include species of *Paraleiocapitella* and exclude species of *Dodecaseta* and *Rashgua*. García-Garza and León-González (2015) suggested a more strict definition that only species with biramous chaetiger 1 were included in the genus. The latest definition proposed by Magalhães and Blake (2017) is in agreement with Green (2002). Under the current scheme, six *Notomastus* species (at species level) were reported to have neuropodial hooded hooks in the last thoracic chaetiger, i.e., *N. americanus* from North Carolina, USA (Day 1973; now synonymous with *N. hemipodus* Hartman, 1945), *N. angelicae* from the Gulf of California (Hernández-Alcántara and Solís-Weiss 1998), *N. daueri* from the Gulf of Mexico (Ewing 1982), *N. mossambicus* from Madagascar (Thomassin 1970), *N. precocis* from California, USA (Hartman 1960), and *N. teres* from waters off New England, USA (Hartman 1965). Ewing (1984) also reported an unnamed species (labelled as *Notomastus* sp. A) with this character.

*Notomastus* species are frequently encountered in Chinese coastal waters. Among the six recorded species to date (Liu 2008), only two species have brief taxonomic descriptions (Sun and Yang 1988), whereas the rest are reported in ecological publications, where only species names are mentioned without any taxonomic descriptions and illustrations. Besides, most of the recorded species have type localities that are distant from China. Therefore, the knowledge of Chinese *Notomastus* species is still poorly understood, and the known records require further examination. Currently, *Notomastus mossambicus* (formerly known as *Paraleiocapitella mossambicus*﻿) is the only species in Chinese waters known to have the last thoracic chaetiger with notopodial capillaries and neuropodial hooded hooks. In this study, a new *Notomastus* species with the same structure of the last thoracic chaetiger was collected from intertidal to shallow subtidal habitats in Xiamen Bay, Fujian Province, China. In addition to the structure of chaetiger 11, the new species resembles *N. mossambicus* in having prostomial eyespots and uniramous chaetiger 1, but it differs from the latter in the structure of the prostomium and epithelial texture. The new species is distinguished from other closely related species by morphological characters and gene sequences. With specimens collected from the identical site in different months and from other localities along the southern coasts of China, the morphology and geographical distribution of the new species are also discussed. A identification key is provided for worldwide *Notomastus* species having the last thoracic chaetiger transitional.

## Materials and methods

### Field sampling

**Figure 1.**
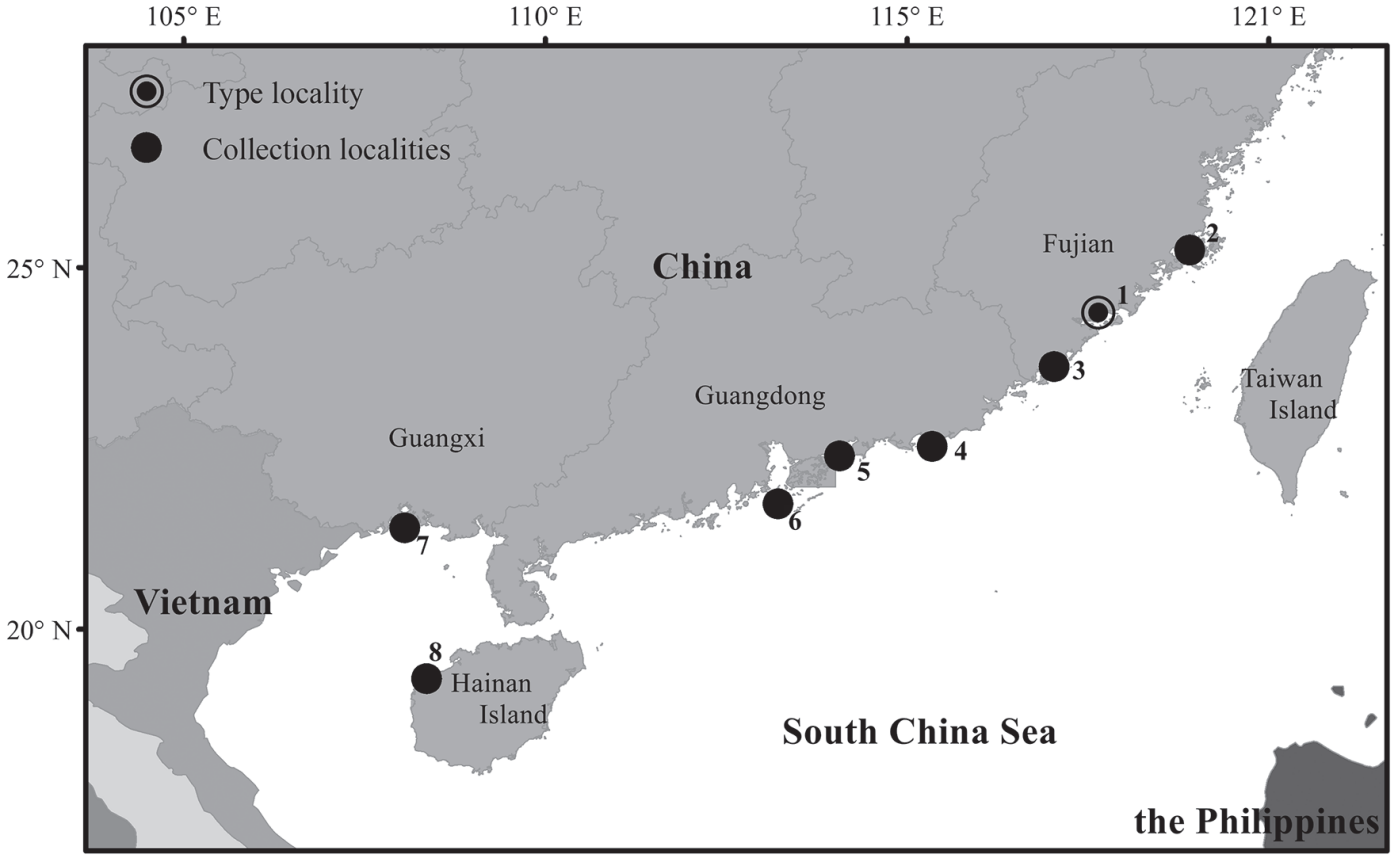
Type locality and collection localities of *Notomastus sunae* sp. nov., **1** Xiamen Bay (Fujian Province) **2** Xinghua Bay (Fujian Province) **3** Dongshan Bay (Fujian Province) **4** Jieshi Bay (Guangdong Province) **5** Daya Bay (Guangdong Province) **6** outside Pearl River estuary (Guangdong Province) **7** Qinzhou Bay (Guangxi Province) **8** water off western Hainan Island.

A collection of over 90 specimens from eight localities along southern China (Fig. 1) was examined in this study. Sediment samples were collected from intertidal or shallow subtidal coastal waters during surveys conducted from 2016 to 2019 using either a grab sampler (subtidal stations) or a sampling frame (intertidal). Sediment samples were washed through a 0.5 mm sieve in the field. Specimens retained were fixed with either 8% diluted formalin in seawater, and later transferred to 70% ethanol, or directly preserved in 95% ethanol.

### Morphological analysis

Specimens were examined using a Leica MZ95 optical stereoscope. Light photographs were taken under a Leica M205A stereoscope equipped with DFC 550 digital camera. The structure of abdominal hooks was observed under Axio Imager Z2 (Carl Zeiss Inc., Oberkochen, Germany) using oil emersion. SEM observations were carried out on a scanning electron microscope (ZEISS SUPRA 55 SAPPHIRE) at Xiamen University, and methyl green staining pattern (MGSP) was used to identify the distribution of glandular areas, both as delineated by Lin et al. (2019). The type material and additional material examined in this study were deposited in the Third Institute of Oceanography, Ministry of Natural Resources, Xiamen, China.

### Molecular analysis

The total genomic DNA was extracted from organisms using Transgen Micro Genomic DNA EE 181 Kit (Transgen, Beijing, China) following the protocol provided by the manufacturer. The PCR reactions were conducted to amplify partial sequences of mitochondrial (COI) and nuclear (18S, H3) genes using primer sets as shown in Table 1. The PCRs (100 μL) contained 73.5 μL of deionized water, 10 μL of TakaRa 10× Ex Taq buffer, 8 μL of dNTP mixture (2.5 mM), 2 μL of each primer (10 μM), 0.5 μL of TakaRa Ex Taq (5 U/μL) and 4 μL DNA template. The thermal cycling conditions were as follows: 95 °C for 240 s; 35 cycles of 95 °C for 45 s, 41 °C (COI) or 43 °C (18S1, 18S2, 18S3, H3) for 60 s, and 72 °C for 80 s; and 72 °C for 7 mins. 5 μL of the resulting PCR products were checked using 1% agarose gel electrophoresis, and the remaining products were purified using a Transgen Quick Gel Extraction EG 101 Kit (Transgen, Beijing, China) following the manufacturer’s protocol. Sequencing of the purified DNA samples was performed at Biosune company (Xiamen, China) with an ABI 3730XL DNA analyzer (Applied Biosystems).

**Table 1.** List of primer sets used for PCR and sequencing in this study.

Gene	Primer name	Sequence (5′ to 3′)	Reference
COI	LCO 1490	GGTCAACAAATCATAAAGATATTGG	Folmer et al. (1994)
	HCO 2198	TAAACTTCAGGGTGACCAAAAAATCA	Folmer et al. (1994)
H3	aF	ATGGCTCGTACCAAGCAGAC	Colgan et al. (1998)
	aR	ATATCCTTRGGCATRATRGTGAC	Colgan et al. (1998)
18S1	F	GCTGTATGTACTGTGAAACTGCG	Song et al. (2018)
	R	GGAATTACCGCGGCTGCTGGCACC	Song et al. (2018)
18S2	F	GTTCGATTCCGGAGAGGGAGCCT	Song et al. (2018)
	R	GTTTCGGCCTTGCGACTATACTT	Song et al. (2018)
18S3	F	ACTGCGAAAGCATTTGCCAAGAGT	Song et al. (2018)
	R	CACCTACGGAAACCTTGTTACGAC	Song et al. (2018)

Obtained sequences were manually checked and assembled into a consensus sequence using the software DNAMAN 8 (Lynnon Biosoft, Quebec, Canada). Eventually, about 650 bp of COⅠ, 1637 bp of 18S, and 316 bp of H3 were successfully amplified in this study. The available sequences of related genera of Capitellidae in GenBank were used in phylogenetic analysis (Table 2). Alignments of the sequences were performed using the MUSCLE algorithm (Edgar 2004) implemented in the software MEGA Ⅹ (Kumar et al. 2018) under default settings. The unaligned sequences and highly divergent regions were removed using Gblocks (Castresana 2000). A maximum likelihood (ML) analysis was conducted in RAxMLGUI 1.5 beta (Silvestro and Michalak 2012) on the concatenated sequence of 18S and H3 genes, using the model GTR+G+I and 1000 thorough bootstrap pseudoreplicates. The tree was edited using FigTree v. 1.4 (Rambaut 2012) and Adobe Photoshop CS5. The aligned and trimmed sequences were used as data sets to generate the interspecific genetic distance using the Kimura’s two-parameter (K2P) model (Kimura 1980) implemented in MEGA X.

**Table 2.** DNA sequences with GenBank accession numbers used in phylogenetic analysis.

**Species name**	**Origin**	**18S**	**H3**
Ingroup			
*Barantolla lepte* Hutchings, 1974	Australia	AB106265	N/A
*Capitella teleta* Blake et al., 2009	Ehime, Japan	LC208027	LC208089
*Dasybranchus caducus* (Grube, 1846)	N/A	AF448153	N/A
*Heteromastus filiformis* (Claparède, 1864)	N/A	DQ790081	N/A
*Mediomastus opertaculeus* Hiruta & Kajihara, 2013	Hokkaido, Japan	LC208046	LC208107
*Notomastus hemipodus* Hartman, 1945	Bamfeld, Canada	HM746728	HM746759
*Notomastus koreanus*, Jeong et al. 2018	Busan, Korea	N/A	MG748699
*Notomastus latericeus* Sars, 1851	Bohuslän, Sweden	AY040697	DQ779747
*Notomastus* sp. 1 ST2018	Tokyo, Japan	LC208047	LC208108
*Notomastus* sp. 2 ST2018	Okinawa, Japan	LC208048	LC208109
*Notomastus* sp. 3 ST2018	off Owase, Japan	LC208049	LC208110
*Notomastus* sp. 4 ST2018	Suou-Nada, Japan	LC208050	LC208111
*Notomastus* sp. 5 ST2018	Okayama, Japan	LC208051	LC208112
*Notomastus* sp. 6 ST2018	Okayama, Japan	LC208052	LC208113
*Notomastus* sp. 7 ST2018	Kagoshima, Japan	LC208053	LC208114
*Notomastus* sp. SIO BIC	Friday Harbor, WA, USA	KF511859	KF511880
*Notomastus sunae* sp. nov.	Xiamen, China	MT055861	MT055862
*Notomastus tenuis* Moore, 1909	N/A	DQ790084	N/A
*Notomastus torquatus* Hutchings & Rainer, 1979	Australia	N/A	AF185258
Outgroup			
*Arenicola marina* (Linnaeus, 1758)	Arcachon, France	AF508116	DQ779718
*Nicomache personata* Johnson, 1901	Hokkaido, Japan	LC006051	LC005496

## Systematics

**Class Polychaeta Grube, 1850**

**Family Capitellidae Grube, 1862**

### Genus Notomastus

Taxon classificationAnimaliaCapitellidaCapitellidae

Sars, 1851

0411A90D-F668-55EE-B958-8AB025EA065F

#### Type species.

*Notomastus latericeus* Sars, 1851

#### Generic diagnosis

(after Magalhães and Blake 2017). Prostomium conical, with or without palpode; eyespots present or absent. Thorax consisted of an achaetous peristomium and 11 chaetigers. First chaetiger uniramous or biramous. Chaetigers 1–11 with only capillaries in both rami or last thoracic chaetiger transitional with notopodial capillaries and neuropodial hooded hooks. Abdominal chaetigers with hooded hooks only. Branchiae present or absent. Genital pores present or absent. Lateral organs present on thorax and abdomen.

### Notomastus sunae sp. nov.

Taxon classificationAnimaliaCapitellidaCapitellidae

B47B8BEE-9D34-5BE6-B07F-0700E3620CC4

http://zoobank.org/1E60348C-F682-457C-862C-D7BED5215024

**Figure 2.**
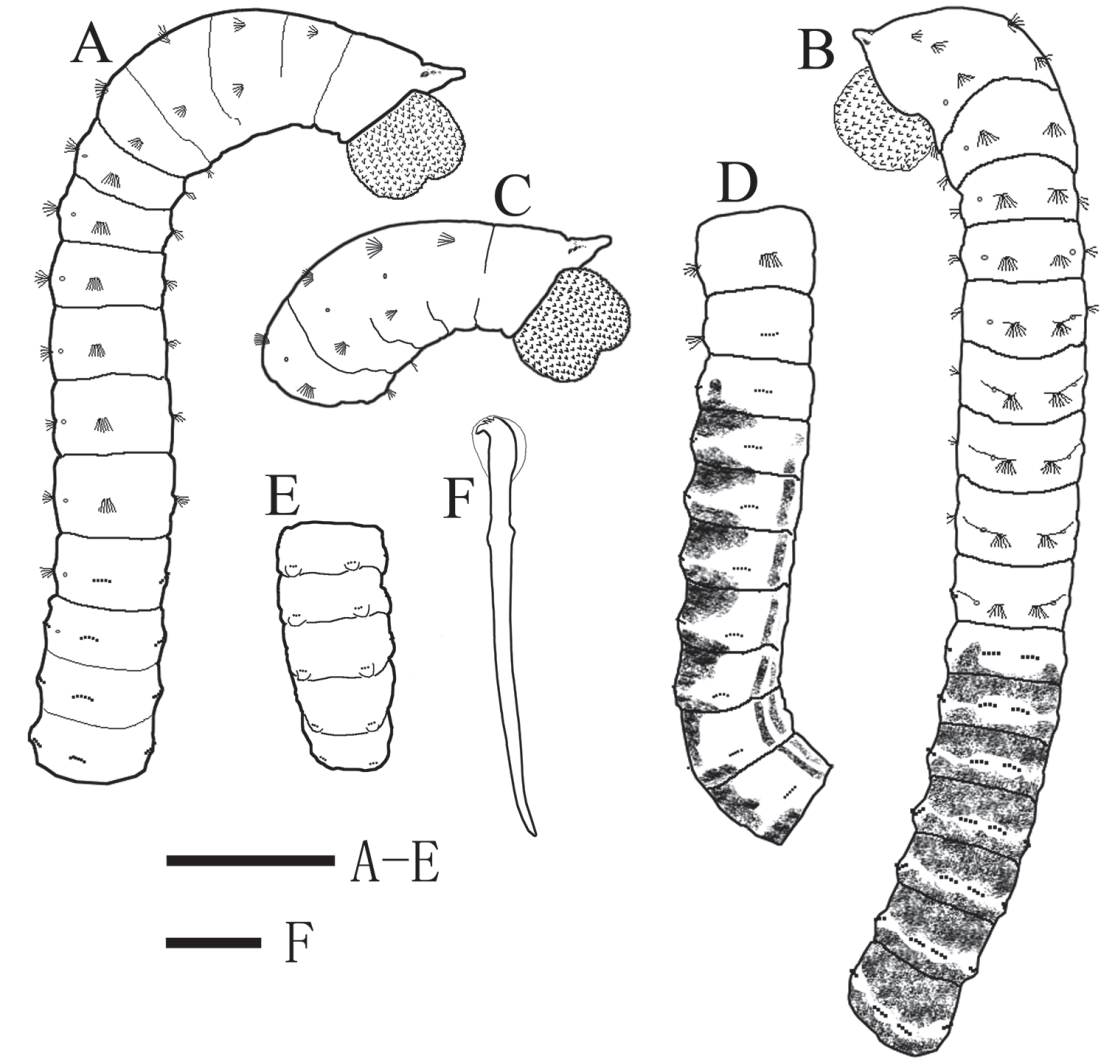
*Notomastus sunae* sp. nov., holotype. **A** thorax and anterior abdomen (14 chaetigers) in ventrolateral view **B** thorax and anterior abdomen (18 chaetigers) in dorsal view **C** anterior end in lateral view, showing eyespots and papillae **D** chaetigers 10–19 in ventrolateral view, showing transition between thorax and abdomen **E** far posterior abdomen in dorsal view, showing notopodia with simple epithelial extensions **F** abdominal hooded hooks. Shading on **B** and **D** indicates methyl green staining. Scale bars: 1 mm (**A–E**); 20 μm (**F**).

**Figure 3.**
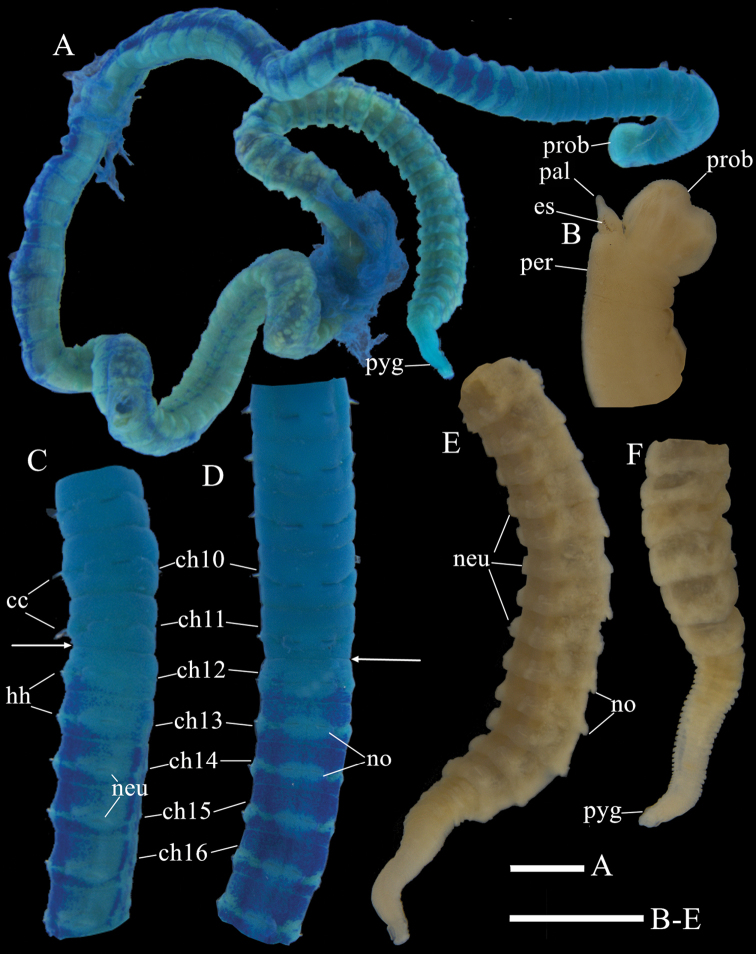
*Notomastus sunae* sp. nov., holotype. **A**MGSP of whole body **B** anterior end in lateral view **C**MGSP of transitional segments (chaetigers 9–17) between thorax and abdomen in ventrolateral view **D**MGSP of transitional segments (chaetigers 7–17) between thorax and abdomen in dorsal view **E** posterior end in ventrolateral view **F** posterior segments near pygidium in dorsal view. Abbreviations: cc, capillary chaetae; ch, chaetiger; hh, hooded hooks; neu, neuropodia; no, notopodia; pal, palpode; per, peristomium; prob, proboscis; pyg, pygidium. Scale bars: 1 mm (**A**); 1 mm (**B–E**).

**Figure 4.**
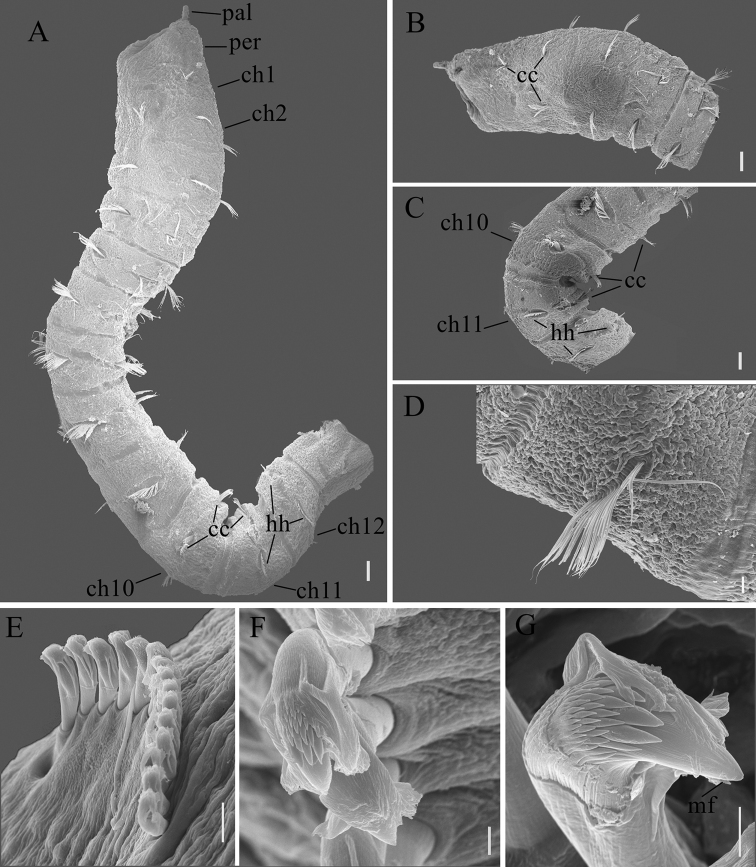
SEM photos of *Notomastus sunae* sp. nov., paratype (TIO-BTS-Poly-115) **A** anterior body in lateral view **B** anterior end in dorsolateral view **C** chaetigers 8–12 in lateral view **D** capillary chaetae **E–G** hooded hooks. Abbreviation: cc, capillary chaetae; ch, chaetiger; hh, hooded hooks; mf, main fang; pal, palpode; per, peristomium. Scale bars: 100 μm (**A–C**); 20 μm (**D**); 10 μm (**E**); 2 μm (**F, G**).

Figures 2A–F , 3A–F , 4A–G

#### Type material examined.

***Holotype***: TIO-BTS-Poly-114 (sta. XM12)–Xiamen Bay, Fujian Province, [24°33'54"N, 118°10'00"E], 6 m, mud, complete, 25 August 2018, coll. Junhui Lin. ***Paratypes***: TIO-BTS-Poly-115–6 specimens, same information as holotype, one mounted on SEM stub; TIO-BTS-Poly-116 (sta. QPW1-4)–9 specimens, Xiamen Bay, [24°27'16"N, 118°10'20"E], intertidal, muddy sand, 23 January 2019; TIO-BTS-Poly-117–9 specimens, 4 April 2019; TIO-BTS-Poly-118–23 specimens, 24 July 2019; TIO-BTS-Poly-119–4 specimens, 13 September 2019; TIO-BTS-Poly-120–16 specimens, 30 October 2019. Specimens (from TIO-BTS-Poly-116 to TIO-BTS-Poly-120) collected from the identical site (QPW1-4) by Junhui Lin.

#### Additional material examined.

TIO-BTS-Poly-121 (sta. XHW04)–3 specimens, Xinghua Bay (Fujian Province), [25°25'55"N, 119°24'16"E], 7 m, mud, 17 April 2019, coll. Zhong Li; TIO-BTS-Poly-122 (sta. DS06)–1 specimen, Dongshan Bay (Fujian Province), [23°48'57"N, 117°31'41"E], 5 m, mud, 26 February 2019, coll. Heshan Lin; TIO-BTS-Poly-123–2 specimens, same location as TIO-BTS-Poly-122, 17 June 2019, coll. Heshan Lin; TIO-BTS-Poly-124–6 specimens, Jieshi Bay (Guangdong Province), [22°45'22"N, 115°47'09"E], 8 m, mud, 19 August 2019, coll. Zhizhong Huang; TIO-BTS-Poly-125–2 specimens, [22°42'40"N, 115°48'10"E], 21 m, muddy sand, 19 August 2019, coll. Zhizhong Huang; TIO-BTS-Poly-126–1 specimen, Daya Bay (Guangdong Province), [22°34'42"N, 114°33'30"E], 12.5 m, mud, 20 February 2016, coll. Junhui Lin; TIO-BTS-Poly-127–2 specimens, outside Pearl River estuary (Guangdong Province), [21°54'49"N, 113°42'15"E], 23 m, muddy sand, 24 October 2019, coll. Zhizhong Huang; TIO-BTS-Poly-128 (sta. GFC-S23)–4 specimens, Qinzhou Bay (Guangxi Province), [21°35'04"N, 108°32'07"E], 7 m, muddy sand, 28 October 2017, coll. Zhong Li; TIO-BTS-Poly-129 (sta. GFC-S11)–1 specimen, [21°37'34"N, 108°38'15"E], 9.5 m, muddy sand, 20 April 2018, coll. Zhong Li; TIO-BTS-Poly-130 (sta. GFC-S23)–1 specimen, same location as TIO-BTS-Poly-128, 19 April 2018, coll. Zhong Li; TIO-BTS-Poly-131 (sta. GFC-S33)–1 specimen, [21°34'31"N, 108°52'42"E], 7 m, muddy sand, 21 April 2018, coll. Zhong Li; TIO-BTS-Poly-132 (sta. GFC-S02)–2 specimens, [21°37'32"N, 108°34'57"E], 12 m, mud, 17 August 2018, coll. Zhong Li; TIO-BTS-Poly-133 (sta. GFC-S19)–2 specimens, [21°31'55"N, 108°34'29"E], 12 m, sand with shell fragment, 17 August 2018, coll. Zhong Li; TIO-BTS-Poly-134 (sta. GFC-S48)–1 specimen, [21°39'29"N, 108°36'47"E], 14 m, mud, 17 August 2018, coll. Zhong Li; TIO-BTS-Poly-135 (sta. CJ03)–2 specimens, off western Hainan Island, [19°27'56"N, 108°49'40"E], 20 m, muddy sand, 22 May 2019, coll. Zhong Li; TIO-BTS-Poly-136 (sta. CJ07)–2 specimens, [19°29'46"N, 108°50'24"E], 18 m, muddy sand, 22 May 2019, coll. Zhong Li.

#### Comparative type material.

*Notomastus hemipodus* Hartman, 1945, holotype: LACM-AHF Poly-414–North Carolina, Bogue Sound, dredged in a few feet of water, 15 June 1940; paratypes: LACM-AHF Poly-415–North Carolina, Bogue Sound, June 1940; LACM-AHF Poly 2667–muddy sand at low tide, June 1940; LACM-AHF Poly 2668–incomplete, muddy sand flats at low water, June 1940; LACM-AHF Poly-2669–incomplete, outer end of Bird Shoal, 18 June 1940, coll. O. Hartman. *Notomastus americanus* Day, 1973, Holotype: USNM 43118–North Carolina, Beaufort, 4 June 1965; Paratype: USNM 43119–North Carolina, Beaufort, 4 June 1965 coll. J. Day.

#### Sequence.

MT055861 (18S, 1637 bp), MT055862 (H3, 316 bp), MT055863 (COⅠ, 650 bp), determined from paratype (TIO-BTS-Poly-118).

#### Description.

Holotype complete with over 100 chaetigers (Fig. 3A), measuring 33.74 mm long by 0.8 mm wide. Paratypes complete or incomplete, ranging from 6.81–43.02 mm long, 0.57–0.90 mm wide for 19–103 chaetigers. Color in alcohol tan (Fig. 3B). Thorax dorsally rounded, ventrally flattened, widest at chaetiger 3. Prostomium conical, with narrow palpode (Figs 2A–C, 3B, 4A, B). Everted proboscis globular, with numerous minute papillae (Fig. 2A–C). Eyespots present on lateral sides of prostomium (Figs 2A, C, 3B). Peristomium achaetous, wider than long, as wide as first chaetiger, but longer (Fig. 2A). Thorax slightly areolated in anterior 4–5 chaetigers, remaining chaetigers smooth.

Thorax consisted of an achaetous peristomium and 11 chaetigers (Fig. 2A, B). Chaetiger 1 uniramous (Figs 2A, C, 4A, B), with capillaries in notopodia only. Chaetigers 2–10 with only capillaries in both rami (Figs 2A, B, 4A, D). All capillaries bilimbate. Chaetiger 11 transitional with notopodial capillaries and neuropodial hooded hooks (Figs 2A, D, 4A, C). Chaetigers 1–4 slightly expanded. Chaetigers 6–10 biannulated with intra- and inter-segmental grooves (more evident in lateral view), wider than long (Fig. 2A, B). Notopodia inserted dorsolaterally in first five thoracic chaetgiers, then notopodia inserted dorsally from chaetiger 6 to posterior thorax (Fig. 2B). Neuropodia ventrolateral. Chaetal fascicles inserted just posterior to midline of thoracic segments (Figs 2A, B, 4A). Notopodia of chaetigers 1–11 and neuropodia of chaetigers 2–10 each with 10–25 capillaries per fascicle; neuropodia of chaetigers 11 with approximately 16 hooks per fascicle. Thoracic hooks of similar shape to abdominal hooks, but shaft markedly longer. Lateral organs conspicuous in thorax and anterior abdomen, located between noto- and neuropodia, closer to notopodia, as small rounded pores (Fig. 2A–C). Genital pores present on intersegmental grooves of between chaetigers 7/8, 8/9, 9/10, and 10/11 on holotype.

Transition between thorax and abdomen marked by change in chaetal arrangement and methyl green staining pattern (Figs 2A, B, D, 3C, D, 4A). First abdominal segment as wide as last thoracic chaetiger, but slightly shorter (Fig. 2A, B, 3C, D). Parapodial lobes reduced in anterior abdomen, well separated (Fig. 2A, B). Notopodial lobes located dorsally (Fig. 2B), close together in anterior abdomen, becoming dorsolateral in posterior abdomen. Neuropodial lobes lateral, separated ventrally. Chaetal fascicles positioned posterior to midsegment in anterior abdomen (Fig. 2B, D), and near posterior edge of segment toward the pygidium (Fig. 2E). In the far posterior, notopodial lobes with a simple epithelial extension (Figs 2E, 3E), broadly-based and rounded-tipped. In anterior abdomen, chaetal fascicles with approximately 10 hooks in notopodia and 16 hooks in neuropodia, decreasing to 6 hooks in notopodia and 10 hooks in neuropodia in posterior abdomen, and to 1–2 hooks in segments near pygidium. Notopodial and neuropodial abdominal hooded hooks of similar shape, with angled node, evident constriction, developed shoulder, posterior shaft longer than anterior one, attenuated to terminal end (Fig. 2F). Hood smooth, slightly longer than wide (Fig. 2F). Abdominal hooded hooks (Fig. 4E–G) with multiple rows of teeth above main fang: 4–5 teeth in basal row, 6–8 teeth in second row, and at least 6 teeth in superior row.

No branchiae observed in abdomen. Regenerated pygidium simple, without anal cirri (Fig. 3E, F)

#### Methyl green staining pattern

(Figs 2B, D, 3A, C, D). Thorax stained with blue with slightly different intensity whereas abdomen stained with very dark blue. From postchaetal area of chaetiger 12, abdominal segments dorsally stained with dark blue, extending ventrallaterally, interrupted by parapodial lobes and lateral organs. Toward posterior abdomen, blue stain on abdominal dorsum faded gradually. From chaetiger 13, abdominal segments with paired stripes of ventral stain with darker intensity, interrupted by intersegmental rings.

#### Distribution.

The new species is widely distributed along the southern coasts of China, from Fujian Province westward to Guangxi Province, and southward to Hainan Province (Fig. 1).

#### Ecology.

The examined specimens were collected from intertidal to shallow subtidal coastal waters (~23 m). Sediment was mainly characterized by mud or muddy sand. The new species is especially abundant in nearshore waters off eastern Xiamen Island, Fujian Province.

#### Etymology.

The species is named after Professor Ruiping Sun, in recognition of her contribution to the study of polychaetes from China Seas.

#### Variation.

Eyespots on prostomium were indistinct in several specimens due to preservation in alcohol. MGSP on chaetigers 11–12 may be different among individuals. Some specimens have darker stain on post-chaetal area of chaetiger 11.

#### Remarks.

As the most species-rich genus of Capitellidae, *Notomastus* has more morphological variability, including variation in the structure of the last thoracic chaetigers. Although it is known that hooks may be replaced by capillaries in the middle-posterior thorax of capitellids during ontogeny (Blake 2000), such as the example of *Heteromastus* (Warren and Hutchings 1994), several authors have confirmed the presence of neuropodial hooks in posterior thorax of some *Notomastus* species even when in adulthood (Ewing 1982; Blake 2000; Green 2002, Magalhães and Blake 2017). For instance, among the 44 examined specimens of *N. angelicae*, Hernández-Alcántara and Solís-Weiss (1998) found that 43 specimens possessed only hooks in the neuropodium of chaetiger 11. Nevertheless, less efforts have been devoted to detecting whether this character change during the development of the specimens. In this study, *Notomastus sunae* sp. nov. specimens were collected from the identical site (sta. QPW1-4) in different months (January, April, July, September, and October). All the 61 specimens uniformly have the last thoracic chaetiger (chaetiger 11) transitional with notopodial capillaries and neuropodial hooded hooks, regardless of body size. Additional specimens from other localities also confirm the similar chaetal structure of chaetiger 11 to the type material. These indicate the stability of this character and that it could be used as an identification tool at the species level.

*Notomastus sunae* sp. nov. is readily distinguished from most congeners by the presence of neuropodial hooks in last thoracic chaetiger. Among the known *Notomastus* species with neuropodial hooks in chaetiger 11, *N. sunae* sp. nov. closely resembles *N. mossambicus* by the presence of uniramous chaetiger 1 and prostomial eyespots, but differs from the latter in that the new species has prostomial palpode and slightly areolated epithelium in anterior thorax, whereas *N. mossambicus* has prostomium without palpode and strongly areolated epithelium in anterior thorax as stated by Thomassin (1970) and Cinar (2005). The new species differs from the geographically close Korean species *Notomastus koreanus* described by Jeong et al. (2018) in that the new species bears eyespots on prostomium, reduced parapodial lobes in anterior abdomen, as well as neuropodial hooks in the last thoracic chaetiger. In terms of the MGSP, *N. sunae* sp. nov. has paired stripes of ventral stain, the feature shared by *N. hemipodus* and *N. koreanus*. However, *N. sunae* sp. nov. has very dark blue stain on abdominal dorsum and extending dorsolaterally, which is distinct from other *Notomastus* species.

Based on morphological description and illustration provided by Green (2002), a *Notomastus* species (labelled as N. near hemipodus) reported from Andaman Sea is closely similar to *N. sunae* sp. nov. in a number of characters: presence of palpode and eyespots on prostomium, uniramous chaetiger 1, slightly areolated epithelium on anterior 4–5 chaetigers, and the MGSP on abdomen which has very dark blue stain on dorsum and paired stripes of ventral stain. Green (2002) mentioned that some specimens had chaetiger 11 transitional with notopodial capillaries and neuropodial hooks, which also agreed with *N. sunae* sp. nov. As the specimens examined by Green (2002) only had anterior fragments (23–37 chaetigers) and lacked ultrastructure of hooded hooks and gene sequences, further comparison is hindered. According to the redescription of *N. hemipodus* by García-Garza et al. (2012), the specimens identified as N. near hemipodus could not belong to *N. hemipodus* in that: 1) they had slightly areolated epithelium on anterior thorax instead of strongly tessellated epithelium as in *N. hemipodus*; 2) they had reduced neuropodial lobes in the anterior abdomen instead of expanded neuropodial lobes as in *N. hemipodus*; 3) they had very dark blue stain on abdominal dorsum instead of moderate green stain as in *N. hemipodus*.

*Notomastus sunae* sp. nov. is commonly collected and abundant in Xiamen Bay, Fujian Province, widely distributed westward to Qinzhou Bay, Guangxi Province, and southward to western Hainan Island, based on the examined material obtained from several localities along southern China. Its specimens are found in great geographical ranges at latitude from 19.5N to 25.5N and at longitude from 108.8E to 119.5E. They prefer to inhabit soft sediments, like mud or muddy sand. So far, this species is found in shallow coastal waters less than 30 m deep.

## Sequences analysis

**Figure 5.**
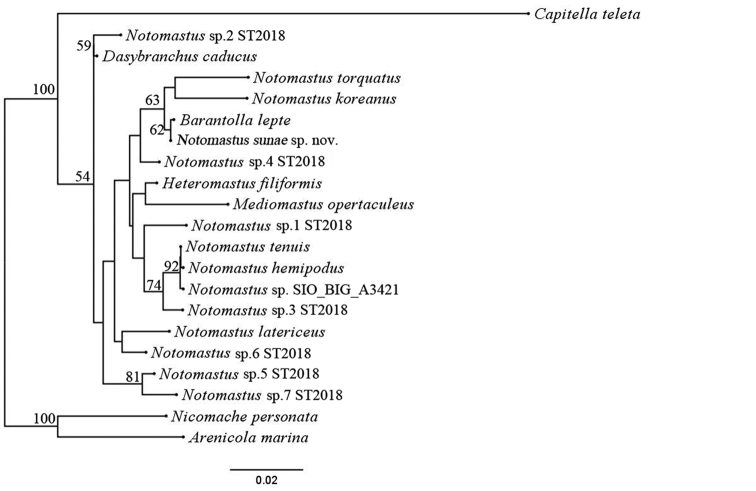
Maximum likelihood tree constructed using the concatenated sequences of *18S rRNA* and *H3*. Only bootstrap support values greater than 50 are shown for each branch. Scale bar represents 0.02 nucleotide substitutions per sequence position.

No identical matches are found for mtCOI, 18S, or H3 of this new species when conducting a GenBank BLAST search. In this study, the maximum likelihood tree (Fig. 5) showed that the new species is sister to *Barantolla lepte* known from Australia with low support value (bootstrap value = 62). The situation might be due to the limited gene sequences included in the analysis, which need further verification. The K2P genetic distances between *N. sunae* sp. nov. and related *Notomastus* species ranged from 20.55% to 74.73% for mtCOI, from 4.107% to 4.109% for 18S rRNA, and from 3.29% to 9.87% for histone H3 (Table 3). For polychaete species, the K2P genetic distance was reported to be 12.3–23.7% among capitellid species (Jeong et al. 2017) and 19.4–26.5% among *Timarete* species (Magalhães et al. 2014) for mtCOI, and 2–9% among cryptic species of *Nereis denhamensis* (Glasby et al. 2013) for histone H3. Therefore, the genetic distance for mtCOI and histone H3 reported in this study, together with distinct morphological characters, indicates that *N. sunae* can be recognized as a new species.

**Table 3.** Pairwise genetic distance (%) base on the K2P model.

	mtCOI	1	2	3		
1	*Notomastus sunae* sp. nov. (MT055863; China)	–				
2	*Notomastus profundus* (KR916899; Porgugal)	74.73	–			
3	*Notomastus koreanus* (MG437148; Korea)	20.55	73.01	–		
	**18S rRNA**	**1**	**2**	**3**	**4**	
1	*Notomastus sunae* sp. nov. (MT055861; China)	–				
2	*Notomastus latericeus* (AY040697; Sweden)	4.11	–			
3	*Notomastus hemipodus* (HM746728; Canada)	4.11	2.03	–		
4	*Notomastus tenuis* (DQ790084; –)	4.11	2.03	0.00	–	
	**Histone H3**	**1**	**2**	**3**	**4**	**5**
1	*Notomastus sunae* sp. nov. (MT055862; China)	–				
2	*Notomastus koreanus* (MG748699; Korea)	3.29	–			
3	*Notomastus torquatus* (AF185258; Australia)	3.72	4.19	–		
4	*Notomastus hemipodus* (HM746759; Canada)	9.87	9.85	9.84	–	
5	*Notomastus latericeus* (DQ779747; Sweden)	9.31	7.83	9.32	7.89	–

## Key to *Notomastus* species with neuropodial hooks in thoracic chaetiger 11

**Table:** 

1	Chaetiger 1 biramous	**2**
–	Chaetiger 1 uniramous, with only notopodium	**3**
2	Prostomium with palpode and eyespots	***N. angelicae* Hernández-Alcantára & Solís-Weiss, 1998**
–	Prostomium without palpode and eyespots	***N. daueri* Ewing, 1982**
3	Prostomium with narrow palpode and eyespots	***N. sunae* sp. nov.**
–	Prostomium without palpode; eyespots present or absent	**4**
4	Prostomium with eyespots; neuropodia of last thoracic chaetiger with hooded hooks	**5**
–	Prostomium without eyespots; neuropodia of posterior two or three thoracic chaetigers with hooded hooks	**6**
5	Epithelium areolated in anterior thorax	***N. mossambicus* (Thomassin, 1970)**
–	Epithelium smooth throughout thorax	***N. americanus*** ^*^ **(Day, 1973)**
6	Neuropodial hooks present in thoracic chaetigers 10–11	***N. teres* Hartman, 1965**
–	Neuropodial hooks present in thoracic chaetigers 9–11	***N. precocis* Hartman, 1960**

## Supplementary Material

XML Treatment for Notomastus

XML Treatment for Notomastus sunae
